# Ecological security assessment at different spatial scales in central Yunnan Province, China

**DOI:** 10.1371/journal.pone.0270267

**Published:** 2022-06-28

**Authors:** Yun Chen, Jinliang Wang, Eldar Kurbanov, Abraham Thomas, Jinming Sha, Yuanmei Jiao, Jingchun Zhou

**Affiliations:** 1 Faculty of Geography, Yunnan Normal University, Kunming, China; 2 Northwest Institute of Eco-Environment and Resources, Chinese Academy of Sciences, Lanzhou, China; 3 Key Laboratory of Resources and Environmental Remote Sensing for Universities in Yunnan, Kunming, China; 4 Remote Sensing Research Laboratory, Center for Geospatial Information Engineering and Technology of Yunnan Province, Kunming, China; 5 Center for Sustainable Forest Management and Remote Sensing, Volga State University of Technology, Yoshkar-Ola, Russia; 6 Council for Geoscience, Pretoria, South Africa; 7 College of Geographical Science, Fujian Normal University, Fuzhou, China; Institute for Advanced Sustainability Studies, GERMANY

## Abstract

Healthy ecosystems are the basis of social and economic development. It is of great significance to conduct ecological security assessments in rapidly urbanization areas. Based on the driving forces, pressure, state, impact, and response (DPSIR) model, five years (1995, 2000, 2005, 2010 and 2015) of remote sensing images, social and economic statistics, and field survey data were used to establish an ecological security assessment index system. The ecological security assessment of central Yunnan Province (CYP) urban agglomeration was conducted at the 1 km × 1 km pixel scale and at the county scale based on the multilevel weighted comprehensive index method. The results showed that: (1) With 2005 as the turning point, the ecological security situation in CYP first decreased and then increased. (2) The ecological security at the county scale was mainly categorized as unsafe. At the pixel scale, ecologically unsafe and relatively unsafe areas were mainly distributed in central, northern, and western CYP. (3) The ecological security deterioration and strengthened spatial distribution differences were caused by habitat fragmentation, different physical geographical conditions, and population agglomeration. These results can provide a basis for the coordination and sustainability of economic development and environmental protection in urban agglomerations with rapid urbanization.

## 1. Introduction

The ecological security of any country is an important cornerstone of its national security system [1, 2]. Ecological security refers to the degree to which human beings are protected from ecological damage and environmental pollution in terms of production, life, and health, which includes basic elements such as drinking water and food safety, air quality, and healthy ecosystems [3]. Currently, traditional security concepts in modern states are being challenged by many ecological threats [4, 5]. With the rapid development of the global economy, numerous ecological problems have occurred, such as resource shortages, environmental pollution, and climate change [6]. Degradation of the natural environment caused by conflicts related to regional resources and global ecological problems can also affect a region’s economic and political security [7]. Thus, the concept of ecological security has become an essential part of national strategies worldwide, along with national defense, economic, and food securities [8]. Ecological security links natural, social, and economic systems which is highly valued by countries globally and has become a key research field in geography and ecology [5, 8, 9].

The ecological security system is a complex artificial ecosystem and includes components related to nature, society, and economy [10]. At present, studies on ecological security have mainly focused on the natural environmental deterioration and human society and economic activities that cause changes in the natural environment [11, 12]. Previous studies have performed ecological risk assessments [13, 14], ecological vulnerability assessments [15, 16], ecosystem health assessments [17], ecosystem service assessments [18, 19], and ecological security assessments [9, 20]. Compared with other types of assessments, ecological security assessments focus on ecological, economic, and social aspects, which are more comprehensive. Ecological security assessment methods mainly include mathematical models [21], ecological models [20], landscape ecological security patterns [22], methods combined with remote sensing [23], and future ecological security scenario simulation and prediction [24, 25]. The assessment of ecological security patterns can accurately identify the ecological risk and sources, and then yield strong policy implications to drive the healthy development of ecosystem [26, 27]. However, related studies still lack an unified assessment index system and criteria, and there are differences among research results [28].

The ecological security pattern in China shows strong spatial variability, and the areas with ecological unsafe are mainly located in the developed coastal areas and the ecological fragile areas of central and west China [29]. Economic development, urban expansion, and land use change are the main reasons for the deterioration of ecological security [29, 30]. With the acceleration of urbanization, the ecological security of cities and urban agglomerations has attracted widespread attention [1, 9, 31]. The increasing urban population, excessive population concentrations, land use changes, development and utilization of resources, and pollutant discharge are important causes of threats to the urban ecological environment [16, 32, 33]. Ecological security patterns based on the relationship between landscape pattern and ecological process provides an effective quantitative framework for identifying land degradation risk in rapidly urbanized areas [26].

According to the “Development Plan of Central Yunnan Urban Agglomeration” [34], the central Yunnan Province (CYP) urban agglomeration is not only the social and economic center of Yunnan Province and an important component of Yunnan’s integration into the Belt and Road Initiative, but also an important part of the national “two horizontal and three vertical” urbanization strategic pattern. The two horizontal parts are the land bridge passage (along a railway from Lianyungang to Urumqi) and the passage along the Yangtze River. The three vertical parts are passages along the coastal zone, Beijing-Harbin and Beijing-Guangzhou railways, and Baotou-Kunming railway. CYP is a key zone of western development and building new support belt for China’s economy based on the Yangtze River. However, rapid economic development has caused serious regional ecological pressure, such as dense populations, transportation infrastructure construction, high development intensity, and water shortages [35, 36]. The ecological security situation has inevitably been strongly impacted by the natural, social, and economic development in CYP.

Previous studies on the ecological security assessment of urban agglomerations have mainly carried out by using socioeconomic statistics to reflect the overall situation of ecological security in large administrative regions [36–39]. However, such studies cannot assess the spatial differences within the region. Consequently, the assessment results of different studies vary considerably [31, 37]. In this context, we carried out multi-scales’ ecological security assessment at the 1 km × 1 km and county scales in CYP. We hypothesized that the ecological security patterns in CYP is unbalanced and changeable. Our goals were to (1) establish an assessment index system suitable for underdeveloped areas, and (2) provide insights into the ecological security situation during rapid urbanization at different spatial scales in CYP. The results can provide a basis for economic development and environmental protection decision-making in CYP and in other regions with rapid urbanization around the world.

## 2. Materials and methods

### 2.1. Study area

**Fig 1:**
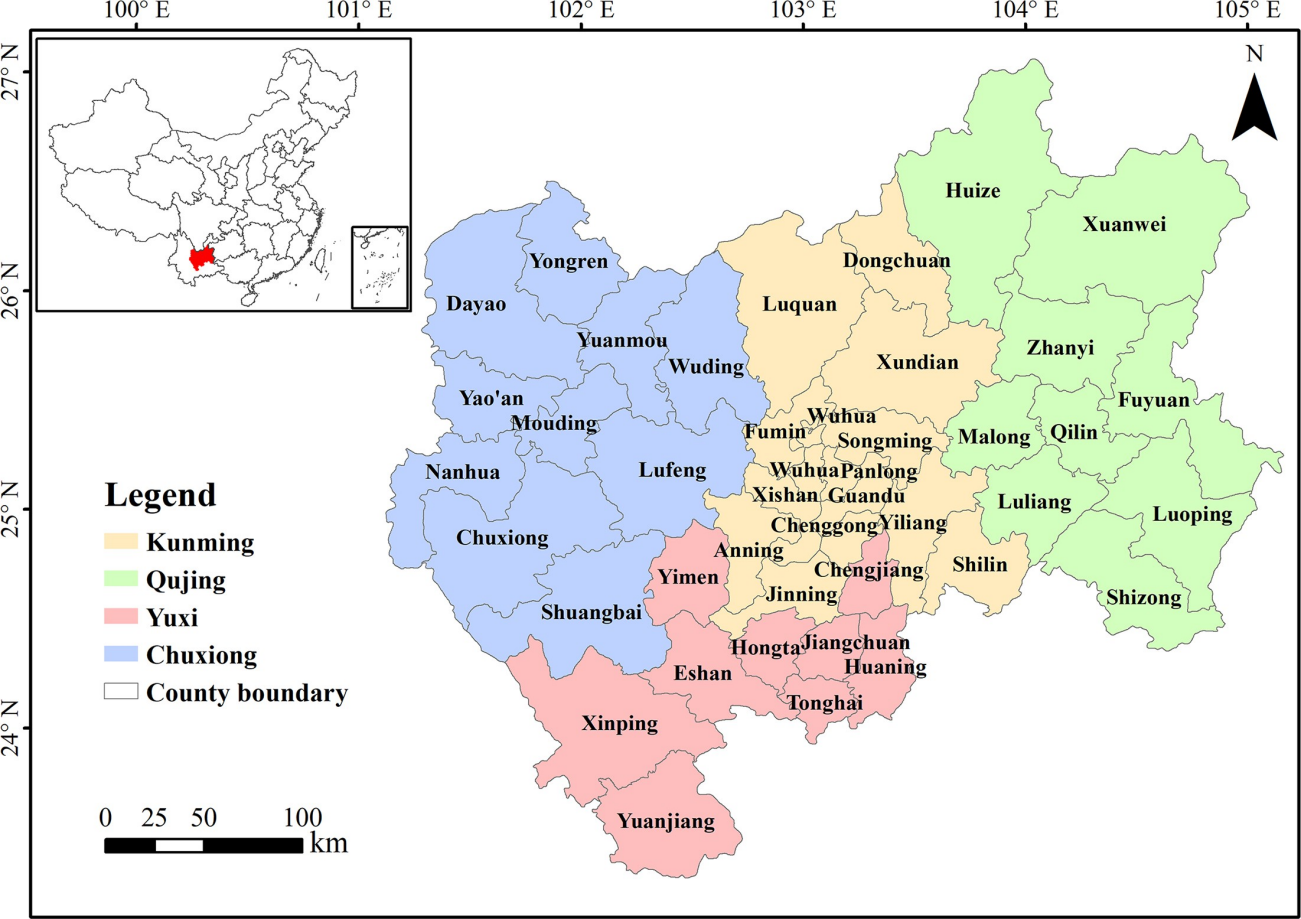
Location of the study area.

CYP is located in the southwestern of China and the central and eastern of Yunnan Province, which is an important urban agglomeration in Southwest China (100°43′E~104°50′E and 23°19′N~27°03′N, Fig 1). This region is located in the upper reaches of the Yangtze River, the Pearl River, and the Red River. The study area consists of four prefectures: Kunming Prefecture, Qujing Prefecture, Yuxi Prefecture, and Chuxiong Autonomous Prefecture. There are 42 counties (district- and county-level cities), with a total area of approximately 9.6 × 10^4^ km^2^, accounting for 24.37% of the total area of Yunnan Province. The climate type of CYP is subtropical plateau monsoon climate. The terrain in CYP is high in the northwest and low in the southeast, with a maximum relative elevation difference of 3920 m. The terrain is dominated by mountains and basins in CYP and the area of plain in CYP is two-thirds of that in Yunnan Province. CYP is also rich in mineral resources, and has most of the mineral resources in Yunnan Province [34]. According to the report of Yunnan provincial government, in 2015, the total population of CYP was approximately 17.81 million, accounting for 37.58% of the total population in Yunnan Province. The gross domestic product (GDP) was 759.4 billion yuan (CNY), accounting for 55.76% of the total amount of Yunnan Province. Additionally, the per capita GDP was 42617 yuan (CNY), which was 1.48 times higher than the average level of Yunnan Province. CYP is the leader in terms of the social and economic development in Yunnan Province and provides important support for the integration of Yunnan into the Belt and Road Initiative. However, compared with China (with 56.1% of the urbanization rates and 49229 yuan (CNY) of the per capita GDP), the development level of CYP (with 46% of the urbanization rates) is still low, which indicated CYP is an underdeveloped region of China. Agricultural production occupies an important proportion of the regional economy of CYP. In addition, CYP has serious ecological problems, such as strong environmental development and utilization and a large resource consumption [31, 35, 40]. Drought, the shortage of water resources, frequent disasters, karst rocky desertification, and soil and water losses in CYP are serious and outstanding, and the ecological security situation is not favorable [35, 40].

### 2.2. DPSIR model and indicator system

The driving forces, pressure, state, impact, and response (DPSIR) model was proposed by the European Environmental Agency (EEA) in 1999 and is a framework to evaluate the index system based on environmental system causality, organizational information, and related indexes [41]. Driving forces (D) describe the factors (such as social and economic developments) that provoke changes in the overall levels of production and consumption and exert pressure on the environment. Pressure (P) indexes describe the potential threat to the environment, such as the release of substances and the use of resources and land. State (S) indexes describe the situation or background value of the environment. Impact (I) indexes describe the impact on the environment exerted by these indicators. Response (R) indexes refer to the positive measures taken by social groups or individuals to prevent, compensate, ameliorate, or adapt to changes in the environment. The DPSIR model was developed based on the pressure, state, response (PSR) model and the driving forces, state, response (DSR) framework [42]. The DPSIR model adds impact factors to the foundation of the PSR and DSR framework. The DPSIR model not only focuses on the factors that cause pressure on the environment but also predicts the environmental impact caused by pressure factors through a causal relationship [42]. Although DPSIR framework has shortcomings [43], it is still an important and effective policy auxiliary tool [44], which has been gradually applied to ecosystem assessments [32].

Previous studies on ecological security assessment in Jinsha River basin and land resources assessment in CYP mainly focused on regional population, economic development, cultivated land, climate change, and vegetation cover [45, 46]. Wu *et al*. [31] bring night light and vegetation productivity factors into the ecological security assessment on pixel scale. Based on the social and economic development of CYP (introduced earlier) and previous studies [31, 45–47], we mainly focused on the meteorological, topographic, agricultural production, economic development, health security, vegetation coverage, and landscape ecology factors, and then established an assessment index system under the guidance of the DPSIR. The indicator system was divided into three levels (target, criterion, and index) comprising 27 indicators, which were divided into positive indicators (i.e., positively correlated with ecological security) and negative indicators (i.e., negatively correlated with ecological security) (Table 1). It’s worth noting that, for example, since CYP is an underdeveloped region of China, the appropriately increasing urbanization rate and economic level will contribute to improve the ecological security (positive correlation). Warming may lead to the strengthening of drought (negative correlation), while increasing precipitation will improve the water resources carrying capacity in CYP (positive correlation).

**Table 1:** Index system of ecological security assessment in central Yunnan Province (CYP).

Target level	Criterion level	Index level	Unit	Data source	Symbol	Weight	Description
*ESI*	Driving Forces (D)	D1: GDP per capita	CNY	Statistics	+	0.0514	Gross domestic product per residential population.
		D2: Annual GDP growth rate	%	Statistics	+	0.0190	The growth rate of GDP in the current year compared with the previous year.
		D3: Urbanization rate	%	Statistics	+	0.0142	The proportion of the urban population to total population.
		D4: Gross domestic product of industrial enterprises above a designated size	Million CNY	Statistics	+	0.0108	The gross product of industrial enterprises with annual revenue of more than 5 million yuan.
		D5: Fixed asset investment amount	Million CNY	Statistics	+	0.0086	The total value of constructed and purchased fixed assets.
		D6: Proportion of the tertiary industry	%	Statistics	+	0.0061	The proportion of the tertiary industry output value relative to the GDP.
	Pressure (P)	P1: Population density	Population km^−2^	Statistics	−	0.0873	The population per area.
		P2: Natural population growth rate	‰	Statistics	−	0.0357	The ratio of the natural population increase within one year to the average population in the same period.
		P3: Fertilizer application amount per unit of cultivated land	kg ha^−1^	Statistics	−	0.0138	Reflects the application intensity of fertilizer.
		P4: Pesticide application per unit of cultivated land	kg ha^−1^	Statistics	−	0.0105	Reflects the application intensity of pesticide.
		P5: Membrane use per unit of cultivated land	kg ha^−1^	Statistics	−	0.0079	Reflects the application intensity of membranes.
	State (S)	S1: Annual average temperature	°C	Observed data	−	0.0186	Arithmetic mean of annual daily average temperature.
		S2: Annual precipitation	mm	Observed data	+	0.0263	The sum of monthly precipitation throughout the year.
		S3: Average slope	°	DEM	−	0.0689	The arithmetic mean of the slope of a unit at different assessment levels.
		S4: Average elevation	m	DEM	−	0.0521	The arithmetic mean of elevation of a unit at different assessment levels.
		S5: Relief degree of land surface	m	DEM	−	0.0950	The difference between the highest and lowest altitudes at different assessment levels.
	Impact (I)	I1: Cultivated land area per capita	ha	Statistics	+	0.0667	Per capita share of cultivated land resources in the region.
		I2: Per capita net income of rural residents	CNY	Statistics	+	0.0091	The income of rural residents after deducting the cost.
		I3: Shannon’s diversity index (SHDI)	NA	Remote sensing image	+	0.0338	The richness and complexity of landscape types and quantity in the region.
		I4: Splitting index (SPLIT)	NA	Remote sensing image	−	0.0260	The degree of patch separation in the region.
		I5: Largest patch index (LPI)	NA	Remote sensing image	+	0.0207	The proportion of the area of the largest patch to the total area.
		I6: Patch density (PD)	NA	Remote sensing image	-	0.1015	The number of patches per area.
		I7: Normalized difference vegetation index (NDVI)	NA	Remote sensing image	+	0.1385	This is related to vegetation coverage.
	Response (R)	R1: Number of health technicians per 10,000 people	Individuals	Statistics	+	0.0164	This reflects the development of social health care.
		R2: Number of beds in health institutions	NA	Statistics	+	0.0116	This reflects the development of social health care.
		R3: Multiple cropping index	%	Statistics	+	0.0043	The ratio of the total sown area of crops to the cultivated land area, which reflects the intensity of development and utilization of cultivated land resources
		R4: Grain yield per hectare	kg ha^−1^	Statistics	+	0.0452	The grain yield per grain sown area, which reflects the productivity of cultivated land resources

*ESI* is the ecological security index; GDP is the gross domestic product; CNY is Chinese Yuan; NA (not applicable) means no unit; + is a positive indicator; − is a negative indicator.

### 2.3. Data sources processing

Remote sensing, meteorological monitoring, and socioeconomic statistics (Table 1) in 1995, 2000, 2005, 2010 and 2015 (which correspond to the Five-Year Plan and the period of rapid urbanization and Grain-for-Green of China), field measurements, and administrative region (.shp format) were used in the present study. Remote sensing data, including satellite images and digital elevation model (DEM), were downloaded from the United State Geological Survey Earth Explorer website (https://earthexplorer.usgs.gov/). The remote sensing images included Landsat 5 TM data in 1995, 2000, 2005, and 2010 and Landsat 8 OLI data in 2015. The path/rows were 128/42, 128/43, 129/41, 129/42, 129/43, 129/44, 130/42, 130/43, 130/44, 131/42, and 131/43. administrative region was derived from Resources and Environment Science and Data Center (https://www.resdc.cn/).

The meteorological data, including annual average precipitation and temperature, were obtained from the meteorological stations and statistics of each county. The socio-economic statistics were obtained from the Yunnan Statistical Yearbook issued by the Bureau of Statistics of Yunnan Province, the China County (City) Social and Economic Statistics Yearbook issued by the National Bureau of Statistics of China, and the statistical yearbook bulletins issued by the Kunming Prefecture, Qujing Prefecture, Yuxi Prefecture, and Chuxiong Prefecture governments. The field investigation data mainly included land use in the study area, which were mainly used to verify the accuracy of land use classification.

Remote sensing image processing included radiometric calibration, atmospheric correction, geometric correction, mosaicking, cropping, and image classification. The maximum likelihood method (supervised classification) combined with manual correction was used in ENVI 5.3 (https://envi.geoscene.cn/) to extract 6 types of land-use classes at the original resolution of 30 m × 30 m: construction land, cultivated land, forest, grassland, water bodies, and bare and snow-covered lands. According to Otukei and Blaschke [48], the unknown measurement vector is assigned to the class in which it has the highest probability of belonging, and the classification formula is shown in Eq (1). The separability of regions of interest (ROI, the sample of image classification) is greater than 1.8. The overall classification accuracy was 78.7%, and the kappa coefficient was 0.74. Then, the landscape pattern and vegetation indexes were calculated based on the land use type map and remote sensing images, respectively. The equations for calculating the landscape pattern indexes and the normalized difference vegetation index (*NDVI*) are shown in Eqs (2−6). Meteorological maps with a resolution of 1 km × 1 km were obtained by interpolation of meteorological station data using the inverse distance weighting method [49]. For the produced maps with a resolution of 30 m × 30 m, in the end, we unified the resolution of these produced maps to 1 km × 1 km by resampling in ArcGIS 10.2 (https://www.esri.com/).

$$D=\text{ln}\left(a_{i}\right)-\left[0.5\text{ln}\left(\left|Cov_{i}\right|\right)\right]-\left[0.5{\left(Y-M_{i}\right)}^{\text{T}}\left(Cov_{i}^{-1}\right)\left(Y-M_{i}\right)\right]$$ (1)

where *D* is the weighted distance, *a*_*i*_ is the prior probability of class *i*, *Cov*_*i*_ is the variance-covariance matrix of class *i*, *Y* is the unknown measurement vector, *M*_*i*_ is the known classes of class *i*, T is transpose.

$$SHDI=-\sum_{i=1}^{n}S_{\text{i}}\text{ln}\left(\frac{A_{\text{i}}}{A}\right)$$ (2)

where *SHDI* is Shannon’s diversity index, *S*_*i*_ is the area index of landscape *i*, *A*_*i*_ is the area of patch *i*, *A* is the total area of the landscape, and *n* is the number of landscape types.

$$SPLIT=\frac{1}{2}\sqrt{n/A}/S_{i}$$ (3)

where *SPLIT* is the splitting index.

$$LPI=A_{max}/A$$ (4)

where *LPI* is the largest patch index and *A*_*max*_ is the area of the largest patch in the landscape.

$$PD=n_{i}/A$$ (5)

where *PD* is the patch density and *n*_*i*_ is the number of *i* patches.

NDVI=(NIR-R)/(NIR+R) (6)

where *NDVI* is the normalized difference vegetation index, which is one of the important parameters reflecting vegetation growth and vegetation coverage, *NIR* is the reflectance of the near-infrared band, and *R* is the reflectance of the red band.

The spatial pattern map of statistics with a resolution of 1km × 1km was generated by two methods: (1) the standardized values (standardization methods is shown in the following section) were assigned to the corresponding land types where the data are generated. In other words, the standardized values of population density and natural population growth rate, which were mainly generated in construction land, were assigned to construction land, whereas other land uses were assigned a value of 0. In the same way, the standardized values of pesticides per unit of cultivated land, chemical fertilizers, plastic mulch film use, multiple cropping index, and grain crop yields were assigned to cultivated land, whereas other land uses were assigned a value of 0. (2) The statistics (i.e., GDP per capita, annual growth rate of GDP, urbanization rate, GDP of industrial enterprises above a designated size, fixed asset investment amount, proportion of tertiary industry, cultivated land area per capita, net income of rural residents, number of health technicians per 10,000 people, and number of beds in health institutions), which reflect the overall situation of socio-economic development in the region or cannot be easily defined which type of land use they are mainly generated from, were used to generate maps with a resolution of 1 km × 1 km by spatial interpolation of the inverse distance weighting method [49]. These values were directly used at the county scale according to the calculation results or statistical data, and then were standardized.

### 2.4. Indicator empowerment and standardization

The analytic hierarchy process (AHP) was used to determine the weights in this paper (Table 1). The AHP combines qualitative analysis with quantitative assessment and divides the problem into several levels. Each level contains several factors that simplify complex problems, which makes the assessment process clear, logical, and systematic. In this study, we divided the assessment system into three levels, the target, criterion, and index levels, and then ranked and graded at each level based on expert knowledge to obtain the weight value. The specific calculation process of AHP can be found in the research conducted by Dos Santos *et al*. [50].

In this study, range standardization was adopted to dimensionalize the estimated indexes at both 1 km × 1 km and county scales. According to the relationship between indexes and ecological security, range standardization can be divided into positive index standardization and negative index standardization (Eqs (7) and (8), respectively):

Standardization of positive indicators:
$$A_{ij}=\left(X_{ij}-X_{min}\right)/\left(X_{max}-X_{min}\right)$$ (7)
(2) Standardization of negative indicators:
$$A_{ij}=\left(X_{max}-X_{ij}\right)/\left(X_{max}-X_{min}\right)$$ (8)

where *A*_*ij*_ represents the standard value of index *j* of sample *i*, the actual value of index *j* of *X*_*ij*_ sample *i*, *X*_*min*_ represents the minimum value of index *j*, and *X*_*max*_ represents the maximum value of index *j*.

### 2.5. Assessment method

The ecological security status in CYP is the result of the comprehensive effects of the mutually affected and mutually constrained subsystems. The ecological security index (*ESI*) was used to measure the ecological security status of CYP by the multilevel weighted composite index method (Eq (9)) [51]. The maps were generated by version 10.2 of ArcGIS software.

$$ESI=\sum_{j=1}^{n}X_{j}\times W_{j}$$ (9)

where *ESI* is the ecological security index, which is equal to the weighted sum of each index in the indicator system. The larger the value of *ESI*, the better the ecological security status. *X*_*j*_ is the standard value of index *j*, *W*_*j*_ is the weight of index *j*, and *n* is the number of indexes.

Based on the actual situation in CYP and relevant studies [46, 52], the natural breakpoint method (which minimizes the differences among the same group of data) [53] was used to determine the *ESI* grading standard at equal intervals (Table 2) to characterize the relative ecological security situation in CYP.

**Table 2:** Grading standard of ecological security in CYP.

Grading	*ESI*	Description
I (Unsafe)	≤0.570	The ecological environment is seriously disturbed and destroyed, the system structure is extremely broken, the system function is seriously degraded, the ecosystem is extremely fragile, the self-recovery ability is extremely low, environmental disasters are highly prone to occur, and the area is highly susceptible to strong interference.
II (Relatively unsafe)	(0.570, 0.595]	The ecological environment is subject to strong interference and destruction, the system structure is relatively incomplete, the system function is incomplete, the ecosystem is relatively fragile, the self-recovery ability is relatively low, and environmental disasters occur frequently.
III (Critical safe level)	(0.595, 0.620]	The ecological environment is in a critical state of ecological security and insecurity, the ecosystem is subject to certain damage, the system structure and function are starting to degenerate, the ecosystem is basically stable, it can withstand interference to a certain extent and has self-recovery ability, but environmental problems are easily triggered after further interference.
IV (Relatively safe)	(0.620, 0.645]	The ecosystem is less subject to interference and destruction, the system structure and function are relatively perfect, and the ecosystem has strong self-recovery ability. The system is relatively stable, environmental problems are not prominent, and it is suitable for human habitation.
V (Safe)	>0.645	The ecosystem is rarely disturbed and destroyed. The structure and function of the system are basically perfect. The system has a strong ability to withstand interference and has strong self-recovery ability. The system is stable, and the environmental problems are not obvious. The system is suitable for human habitation.

## 3. Results

### 3.1. Pixel-based assessment results

**Fig 2:**
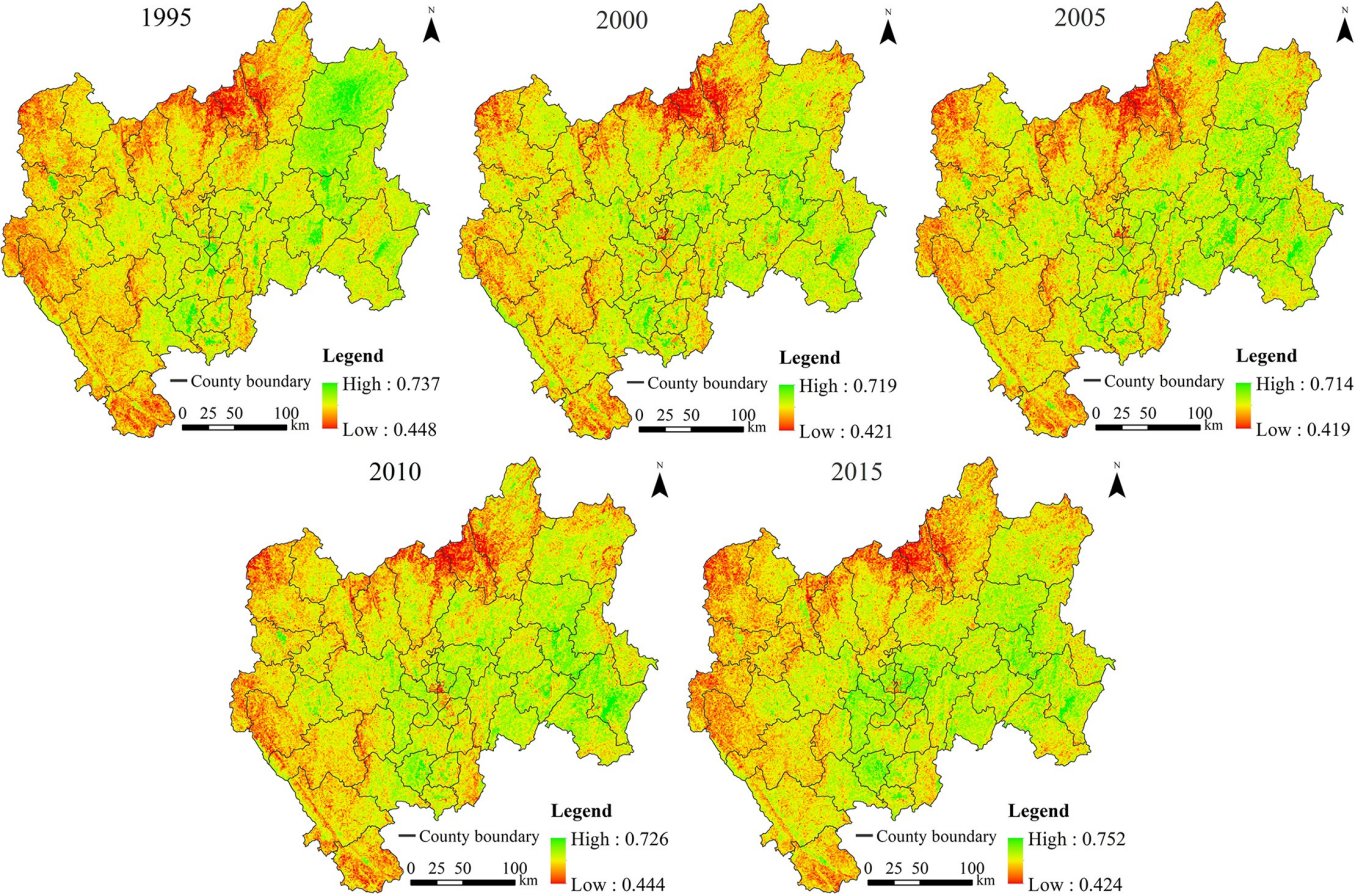
Distribution of the pixel-based ecological security index (*ESI*) in central Yunnan Province (CYP).

**Fig 3:**
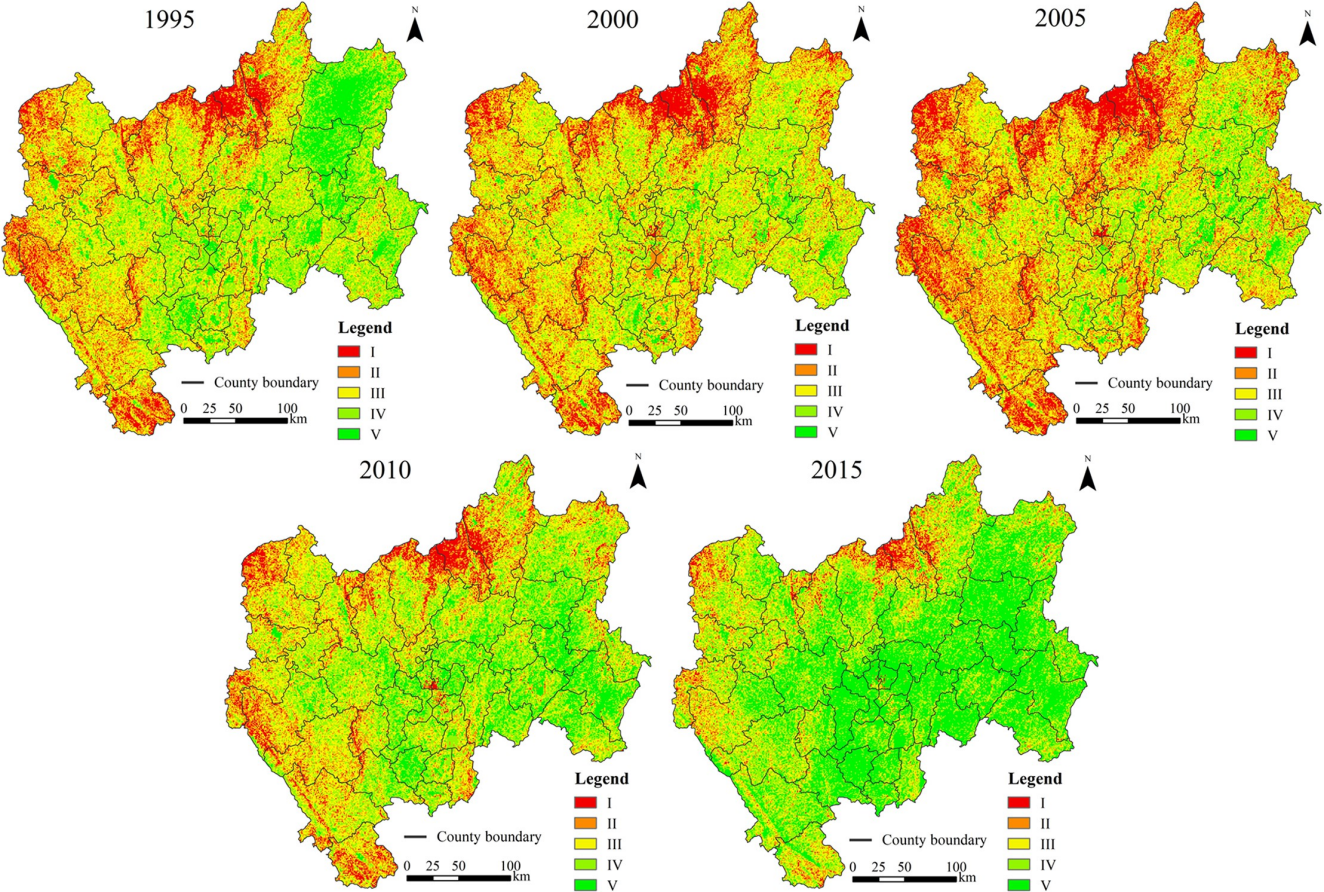
Distribution of ecological security levels in CYP at the pixel level.

The ecological security status and statistical results from CYP were obtained based on pixel-scale data (Figs 2 and 3 and Table 3). In 1995, 2000, 2005, 2010, and 2015, the average *ESI* values were 0.613, 0.603, 0.600, 0.616, and 0.635, respectively. Table 3 showed that in 1995, 2000, 2005, and 2010, the critical safe level of ecological security in CYP accounted for the highest proportion, with all values greater than 34%. The relatively unsafe and unsafe categories in 1995, 2000, 2005, and 2010 together accounted for 25.54%, 34.40%, 42.40%, and 21.50% of the total area, respectively. The summed values of the safe and relatively safe areas in 1995, 2000, 2005, and 2010 were 38.48%, 24.04%, 19.94%, and 44.21%, respectively. In 2015, the region was mainly in a safe and relatively safe state, accounting for 70.22% of the area, and the areas classified as unsafe and relatively unsafe accounted for 9.66%. In general, the overall trend of the *ESI* could be divided into two stages. (1) From 1995 to 2005, the *ESI* was decreased, and the average *ESI* declined by 0.013. In 2005, the lowest index value of 0.419 was observed among the five periods. The proportion of safe and relatively safe areas decreased by 18.54%. (2) From 2005 to 2015, the *ESI* was increased, and the average *ESI* increased by 0.035. In 2015, the highest index value of 0.752 was observed among the five periods, and the proportion of safe and relatively safe areas increased by 50.28%. In general, the ecological security situation in CYP showed a trend towards a safe state.

**Table 3:** Proportions of ecological security levels in CYP at the pixel level (%).

Year	I	II	III	IV	V
1995	6.63	18.91	35.98	27.46	11.02
2000	9.32	25.08	41.56	20.82	3.22
2005	12.62	29.78	37.66	15.74	4.20
2010	6.20	15.30	34.29	31.15	13.06
2015	2.15	7.51	20.12	33.12	37.10

In 1995, the areas with ecologically unsafe and relatively unsafe levels were mainly located in northern, western, and southwestern CYP, including the counties of Huize, Dongchuan, northern Xundian, Fumin, Luquan, Wuding, Dayao, Yao’an, Nanhua, Luding, Chuxiong, Shuangbai, Xinping, Yuanjiang, and western Yimen. These places are typical dry and hot valleys areas in southwest China. The areas with a safe level were mainly distributed in eastern CYP, including Xuanwei, Zhanyi, Qilin, central Luliang, central Luoping, and Hongta counties. The areas with a critical safe level and relatively safe level were dispersed in other areas of the study area. In 2000 and 2005, the ecological security showed obvious degradation. The safe and relatively safe levels in the east had degraded to unsafe or relatively unsafe levels. The unsafe levels in the north were particularly prominent. The critical safe level in the north, west, and southwest gradually evolved into unsafe or relatively unsafe levels, and the state of relatively unsafe area worsened. In 2010 and 2015, the ecological security situation improved significantly, and the proportion of unsafe and relatively unsafe areas decreased significantly. In 2015, the areas with unsafe and relatively unsafe levels were mainly located in northeastern CYP (including the northeast of Xuanwei County, the north of Dongchuan and Luquan counties, and the vicinity of Wuding and Yuanmou counties), western CYP (Dayao, Nanhua, Chuxiong, and parts of Mouding and Yao’an counties), and Yuanjiang County in southwestern CYP. In addition, the areas of safe and relatively safe levels expanded significantly, such as in central, southern, and eastern CYP.

### 3.2. County scale-based assessment results

**Fig 4:**
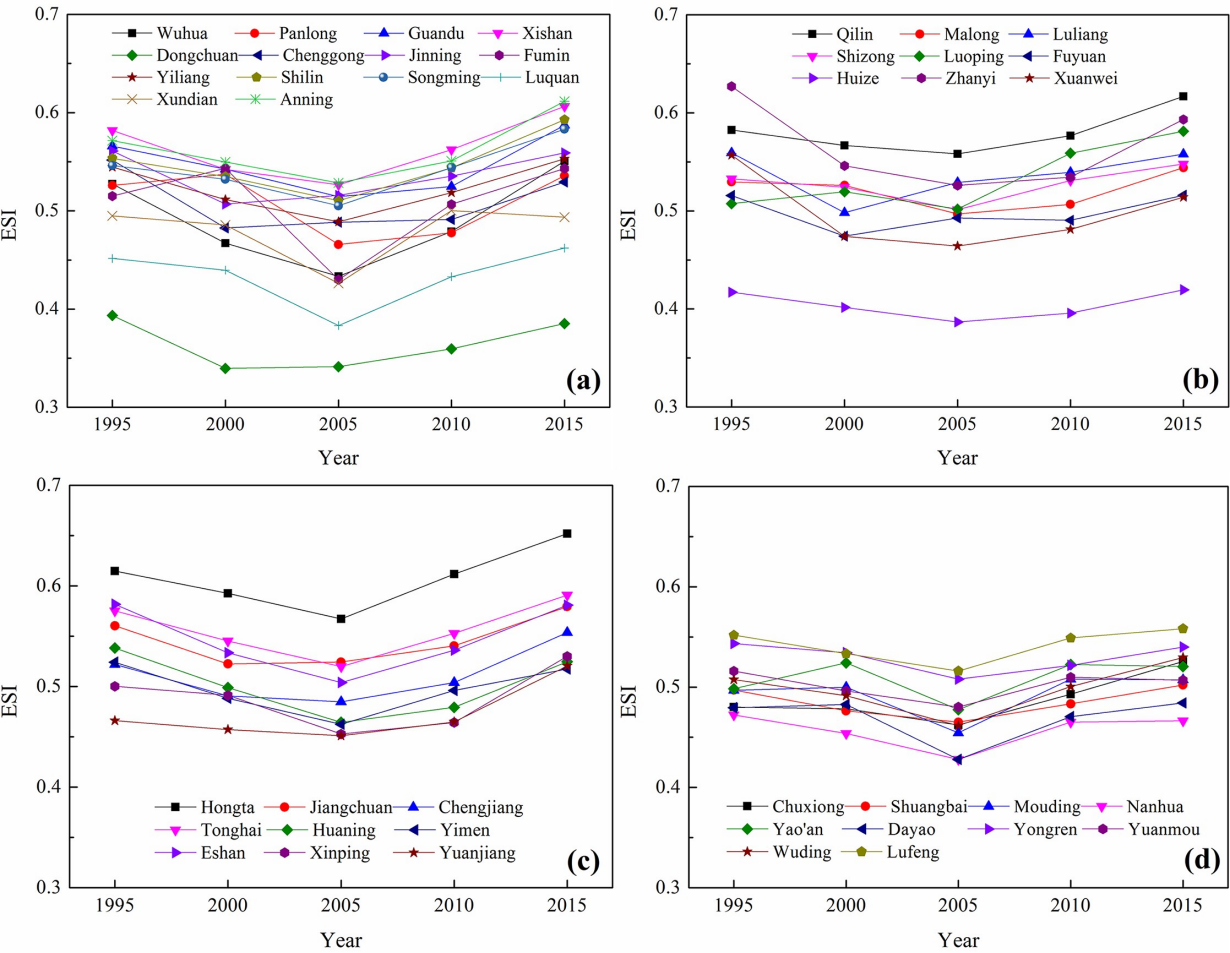
*ESI* values and change trends in CYP at the county level. (a) Kunming Prefecture, (b) Qujing Prefecture, (c) Yuxi Prefecture, and (d) Chuxiong Prefecture.

**Fig 5:**
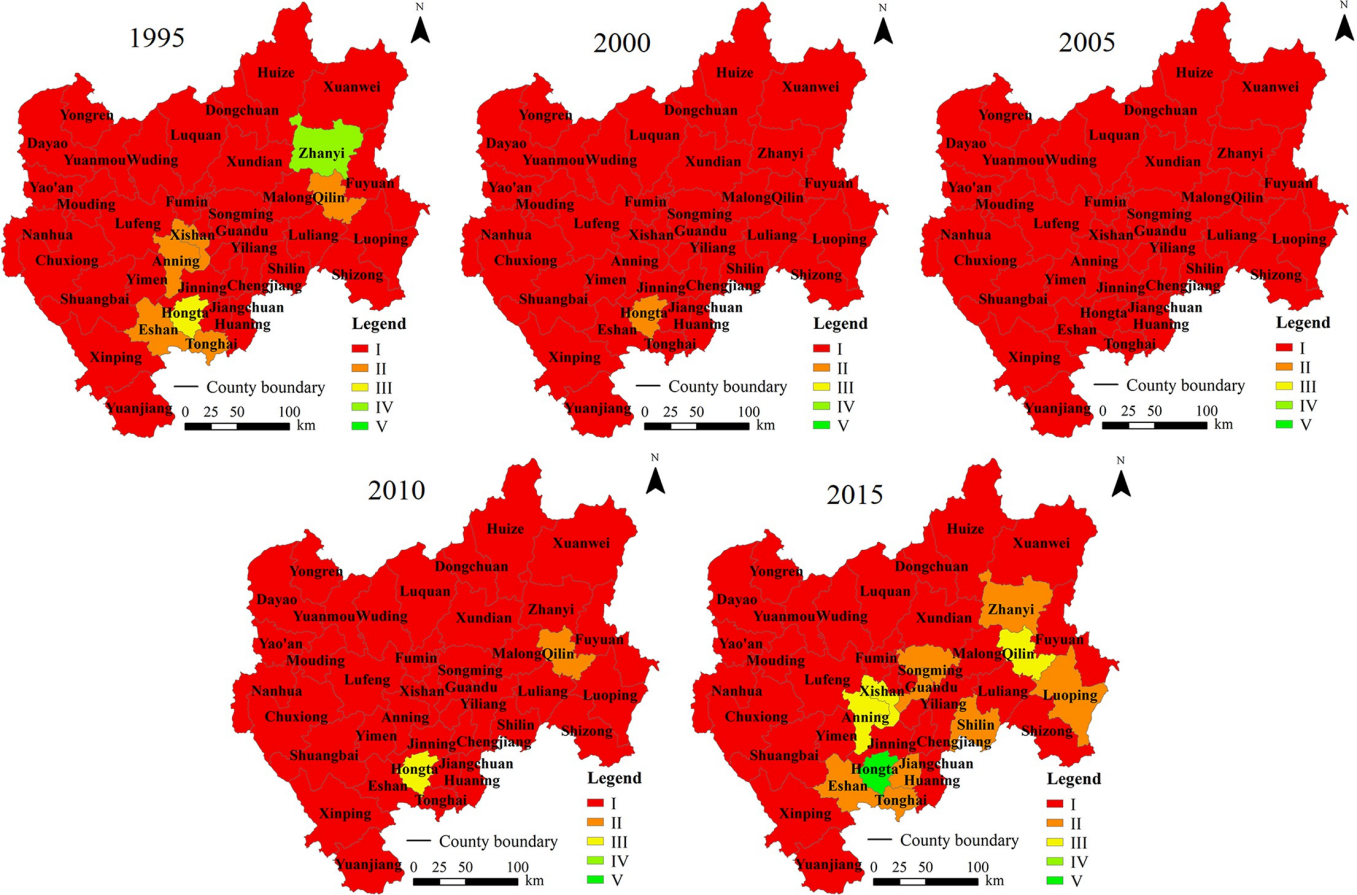
Distribution of ecological security levels in CYP at the county level.

The ecological security status and statistical results of CYP were obtained based on county-scale data (Figs 4 and 5). At the county scale, the average *ESI* values in 1995, 2000, 2005, 2010 and 2015 were 0.527, 0.503, 0.479, 0.508, and 0.540, respectively. As shown in Fig 4, the trend of the *ESI* in CYP was similar to that at the 1 km × 1 km pixel scale, showing a trend of decreasing first and then increasing. Most of the districts and counties had the lowest values in 2005. In *ESI* declining phase from 1995–2005, Zhanyi District experienced the largest decline, and the *ESI* decreased by 0.101. In the increasing phase from 2005–2015, the *ESI* in Wuhua District increased the most, by 0.117. In addition, special attention must be paid to the *ESI*s of 13 districts and counties (Dongchuan, Chenggong, Jinning, Xundian, Huaning, Lushan, Yimen, Luliang, Zhanyi, Xuanwei, Nanhua, Yongren, and Yuanmou) in 2015, which were lower than those in 1995, and the reduction of *ESI* ranged from 0.001 to 0.043. This result showed that the ecological security status of these 13 districts and counties had deteriorated over the past 20 years. Therefore, we should pay more attention to strengthening ecological environmental protection in these areas in the future.

Based on the ecological security classification criteria, the *ESI* at the county scale was divided into five levels, and the patterns were shown in Fig 5. Fig 5 showed that the ecological security in CYP at the county scale was mainly at the unsafe level, indicating a poor ecological security situation. At the county scale, the change in the spatial pattern of ecological security in CYP was consistent with that at the pixel scale. That is, from 1995 to 2005, the number of counties with unsafe ecological situations increased until the ecological situations of all counties became unsafe. From 2005 to 2015, the ecological security situation in CYP improved. By 2015, one county’s ecological security situation was at the safe level. Nevertheless, the ecological security of 38 counties was at unsafe and relatively unsafe levels.

## 4. Discussion

At present, there are no uniform standards for the assessment index system and the grading criteria for ecological security, which leads to differences in the evaluation results among different studies. The conceptual framework models commonly used to construct ecological security assessment index systems include the PSR [54, 55], DSR [56] and DPSIR models [32, 47]. The DPSIR model is an improvement over the PSR and DSR frameworks, inheriting the advantages of the PSR and DSR models while effectively avoiding their shortcomings [41]. Based on the DPSIR model, natural geographical indexes and landscape ecological pattern indexes, combined with social and economic indexes, were selected in this research. The constructed index system can reflect the ecological security status of the study area.

### 4.1. Overall situation of regional ecological security

Previous studies have shown that the overall situation of ecological security in longitudinal range-gorge region of Yunnan Province is good and the areas with high comprehensive index values are mainly located in western Yunnan Province, whereas the areas with poor ecological security are mainly located in central and eastern Yunnan Province, including Kunming Prefecture, Yuxi Prefecture, and Chuxiong Prefecture [39]. In 2008, the ecological situation in CYP was decreased [37, 38]. The results of previous research in the Jinsha River basin in CYP indicated that the ecological security status was extremely unsafe and exhibited decreasing fluctuations [46]. Li [45] suggested that the land carrying capacity of CYP was at intermediate and low levels, with unevenly patterns in space and time series. In terms of water resources, the ecological deficit in CYP was constantly expanding, and the imbalance between supply and demand made it difficult to meet the needs of socioeconomic development [36]. Increased ecological risk, such as the decline in ecosystem services and habitat fragmentation, is also a key problem in the development of Chinese cities with rapid urbanization [1, 57]. In summary, these results of the increasing contradiction between the industrial economy and ecology in CYP and the large spatial difference are basically consistent with present study.

In summary, the spatial pattern and change trend of ecological security were basically consistent with previous study [31]. In other words, the ecological security in CYP is generally at a low level, and the regional development is unbalanced. However, at the county scale, the ecological security differed between present study and Wu *et al*. [31], mainly because the assessment criteria and index system are different. Wu *et al*. [31] mainly researched the pressure on ecosystems and the ability of ecosystems to withstand pressure. Based on the actual situation in CYP, besides the pressure indexes, this study also considered indexes, such as the socioeconomic driving force, the natural state, the impact of pressure, and the response to environmental changes. Compared with Wu *et al*. [31], our results not only identified areas with low ecological security around the urban built-up areas, but also identified high ecological risk areas in dry and hot valleys and disaster-prone areas. The results can provide a basis for ecological protection in CYP. The assessment system and methods can also provide a reference for similar regions with relatively poor economic development levels but rapid urbanization. However, it is worth noting that for different regions, the indicators should be adjusted to local conditions. For example, for resource-based cities, it is important to consider factors related to resource development and utilization, while more attention should be paid to factors related to industrial development, energy consumption, and waste emissions for industrial cities.

### 4.2. Temporal and spatial changes of ecological security

In terms of the change trends, taking 2005 as a turning point, the ecological security of CYP first decreased and then increased. Through in-depth analysis of the assessment results of each subsystem, we believe that there are three main reasons for the change of ecological security in CYP: (1) the population growth rate slowed down. The average natural population growth rate dropped from more than 8% in 1995–2005 to 5% in 2005–2010. (2) The economy of CYP had developed rapidly from 2005 to 2015, and the average per capita GDP was three times of that from 1995 to 2005. In particular, the substantial improvement of rural economy had improved agricultural production technology, which had a positive impact on agricultural production and social security. (3) Vegetation destruction and land use change. The most prominent was the "U" type change trend of NDVI from 1995 to 2015. Other reasons include the "U" type change of precipitation and the obvious warming in CYP from 1995 to 2005. Previous studies suggested that the large-scale occupation of cultivated land, destruction of vegetation, fragmentation of habitats, and degradation of ecosystems were the most direct threats to ecological security in urban agglomerations [1, 57].

In terms of the spatial distribution, the assessment results based on the 1 km × 1 km pixel scale indicated that the areas with poor ecological security were mainly located in northern, western, and eastern CYP and the urban built-up areas of central Kunming Prefecture. These areas in northern CYP (e.g., Dongchuan, Luquan, and Yuanmou) are located in the dry and hot valleys of the Hengduan Mountains, with high elevations, high relief amplitude, frequent droughts, and ecologically fragile [40, 58]. Climate, topography, soil, and runoff factors lead to low vegetation coverage and bare surface in dry-hot valley areas [59]. Thus, the main driving forces of ecological security in north CYP were the natural factors. The previous study reported that the poor ecological security of Kunming City was also located in urban built-up area due to the continuous human activity pressures on ecosystem [31], which is consist with present study. In present study, in the urban built-up area, such as Wuhua, Panlong, and Xishan counties in central CYP, the population density was high, and the pressure of human activities had a negative impact on resources and environment. The main driving forces in these areas with rapid urban expansion were population size and economic factors. The vegetation coverage and biodiversity are generally high in west CYP. However, we found that the overall ecological security in west CYP was poor. The main reason is that this area is the transition area between Hengduan Mountain and Yunnan-Guizhou Plateau, with large topographic relief, less cultivated land resources, and poor social and economic development [60]. To increase agricultural output, large number of pesticides and chemical fertilizers are applied and the degree of land reclamation is high [60]. In contrast, we found that the ecological security situation was good in the mountainous area dominated by Ailao Mountain with high vegetation coverage at the edge of west CYP (Fig 3). We also found that the ecological security in parts of eastern (where the rocky desertification areas located) and central CYP was good, especially it has improved strongly after 2005. The main reasons may be that (1) this area has flat terrain and high resource and environmental carrying capacity, and the areas with good ecological security are mainly located in the plain of Yunnan-Guizhou Plateau [61]; (2) these areas are economically developed, which is conducive to improving ecological security, such as the effective rocky desertification control measures carried out around 2005 [62].

However, there were large differences between the 1 km × 1 km pixel and county scales. The ecological security situation at the county scale was mainly at the unsafe level. At this scale, ecological security showed great complexity and “barrel effect”. Ecological security is affected by natural, economic, and social factors, and is more strongly affected by the worst factors in the region. It is usually easier to obtain a lower score at the county scale when a single evaluation index is used, resulting in *ESI* values lower than those at the pixel scale. For example, the amplitude of relief (negative indicator) at the county scale represents the difference between the maximum and minimum elevations in the whole county and is usually higher than that at the pixel scale, resulting in a lower score. Due to habitat fragmentation, the largest patch index (*LPI*) at the county scale is generally lower than the *LPI* at the pixel scale because the area of the largest patch is still much smaller than the county area. In addition, the scores of indexes that depend on average values are usually at the regional average level, such as the *NDVI*. Thus, there are differences in the influencing factors of ecological security on different scales, which indicated a scale effect. The overall situation of regional ecological security is reflected at the county scale, while the spatial differences within the region are highlighted at the pixel scale.

In summary, we concluded that the change of ecological security in CYP was the result of the interaction of the natural and human factors, but dominated by human factors. Natural driving factors included the relatively arid climate conditions, low vegetation coverage, and disaster-prone terrain conditions. The human driving factors were as follows: (1) the increasing population size and the excessive concentration of the population and (2) the change in land use and the destruction of natural vegetation, which led to habitat fragmentation and the decreasing vegetation coverage.

### 4.3. Implication and improvement of ecological security assessment

Ecological security assessment is significant in decision making. According to the assessment results, the situation of ecological security can be improved through the following measures: (1) control the size of cities. The population of CYP accounts for 38% of total in Yunnan Province, indicating a high population density, especially in central CYP. As the economic center of Yunnan Province, CYP will attract more people. Sub economic centers should be developed based on the existing main centers, and more attention should be paid to the coordinated development of small- and medium-sized cities to reduce the resource pressure caused by urbanization. In addition, urban expansion should be planned reasonably to avoid encroaching on high-quality farmland and biological habitats. (2) Vegetation restoration and disaster management measures are needed to reduce soil erosion and increase vegetation coverage in the dry and hot valleys in north and west CYP. Returning farmland to forest and ecological migration may be effective ways to reduce the interference of human activities in this area. In addition, engineering measures should be taken to improve the ability of disaster prevention and control. (3) In eastern and southern CYP where the ecological environment is better, more nature reserves should be set up and the punishment for ecological damage should be strengthened.

The results of different scales reflected not only the overall status of regional ecological security, but also the spatial differences of ecological security within the region, which can provide a basis for decision making regarding regional economic development and environmental protection. However, there are still some inevitable weaknesses in our research. Although the overall situation of ecological security in CYP has improved over the past 20 years, the continuous impact of climate change on ecological security needs to be further researched. For example, the redistribution of water resources and the potential impact on biodiversity and disaster risk caused by global climate change are crucial to ecological security in CYP. In addition, it was difficult for us to obtain statistics at the township scale, pollutant emissions, and environmental quality in the study area. These factors are also important for ecological security. These weaknesses will be an important part of our future research.

## 5. Conclusions

Located in Southwest China, CYP plays an important role in national strategy and ecological functions. It is of great significance to conduct ecological security assessments of this region. Based on the DPSIR model, an assessment index system based on the DPSIR framework was constructed. The ecological security assessment of CYP was carried out at the 1 km × 1 km pixel and county scales. The results confirmed our hypothesis and showed that although the ecological security status fluctuated from 1995 to 2015, the overall ecological security situation was still not favorable. First, the overall situation of ecological security was low at both scales. Second, there are large differences within the region, and ecological security was imbalanced at the pixel scale. In general, the ecological security in southern and eastern CYP was better than that in western and northern CYP at the pixel scale.
